# Stakeholder bias in best practice advisories: an ethical perspective

**DOI:** 10.1093/jamiaopen/ooaa018

**Published:** 2020-06-28

**Authors:** Aaron Baird, Bryan Kibbe, Jason Lesandrini

**Affiliations:** o1 Institute of Health Administration, and Department of Computer Information Systems, Robinson College of Business, Georgia State University, Atlanta, Georgia, USA, and; o2 Ethics Department, WellStar Health System, Marietta, Georgia, USA

**Keywords:** best practice advisories, ethics, decision support systems, practice guidelines, technology, stakeholder biases

## Abstract

Clinicians are increasingly being asked to heed and follow the guidance provided by “best practice advisories.” Such advisories, often in the form of electronic reminders or alerts, are meant to increase the efficiency and effectiveness of evidence-based medical practice. However, we argue that best practice advisories can sometimes be infused with stakeholder bias, even if inadvertently. We specifically argue that best practice advisory biases can occur when an advisory is not oriented to benefit patients *at least as much* or more than other stakeholders. To address this issue, we put forth the perspective that ethical consideration of biases is especially important in best practice advisory design and revision processes.

## LAY SUMMARY

Best practice advisories are electronic alerts received by clinicians designed to influence clinical actions and guide clinical processes. While such best practice advisories can help clinicians provide more efficient and effective care, it is also possible that biases might be present in best practice advisories. For instance, best practice advisories may be created that result in over prescription or overuse of medical services or devices, perhaps unbeknownst to the recipient of the advisory. We suggest that stakeholder bias, such as bias from hospitals, pharmaceutical companies, or doctors, can be present in such advisories. We argue that reduction or mitigation of stakeholder biases, even if such biases are inadvertently present, requires deliberate ethical considerations during best practice advisory design and review processes.

## THE POTENTIAL FOR STAKEHOLDER BIAS IN BEST PRACTICE ADVISORIES

Best practice advisories are a form of clinical decision support typically seen within an electronic health record (EHR) or closely related system.^1^ These pop-up style alerts or messages are designed to remind, guide, and sometimes require clinical actions. Best practice advisories are assumed to be designed with the best of intentions, typically to encourage or require situationally relevant evidence-based practices. Unfortunately, though, conflicts of interest can appear in best practice advisories, often unbeknownst to the recipient of the advisory. For instance, Practice Fusion, Inc., a cloud-based EHR vendor, recently entered into a $145 000 000 settlement with the US attorney for soliciting a “nearly $1 million payment from a company identified only as ‘Pharma Co. X’ in exchange for creating an alert in Practice Fusion’s EHR system. The alert would cause doctors to write more prescriptions for extended-release opioids than were medically necessary.”^2^ Such a case makes us wonder if bias in best practice advisories is more prevalent than we want to admit. For instance, hypothetically speaking, what if a best practice advisory notifies a hospitalist that an admitted patient was admitted for the same issue in the past 30 days? Is this best practice advisory implicitly encouraging the hospitalist to find an alternative admitting diagnosis to avoid the potential readmission penalty or, rather, is this advisory simply helping the hospitalist understand that additional information may be available that may not have been considered without this advisory?^3^ Or, when multiple courses of action are available that have potentially similar outcomes,^4^ will only the options that maximize business interests, such as referral to a preferred entity, be presented in the advisory?^5^

We argue that like many technologies, best practice advisories and the clinical decision support systems that underlie them are not wholly neutral tools. For example, social media sites and even search engines are often designed to ultimately sell advertisements, which may mean that rather being designed to be neutral communication tools or information retrievers, they are actually designed to persuade or manipulate human behavior (eg, click on more content and advertisements).^6^ Technologies are frequently designed with specific purposes in mind, and thereby are engineered or configured to encourage some actions while discouraging or prohibiting other actions.^7^ This is perfectly understandable, reasonable, and useful—up to a point. However, as Verbeek^7^ and Winner^8^ have observed, it is important to be conscious of the sometimes subtle ways in which underlying value commitments, moral sensitivities, and stakeholder interests can become baked into a technology during the design process, so to speak, and then exhibit tremendous ethical implications and consequences. For example, if a building architect is ignorant about or insensitive to the needs of persons with physical handicaps, then he or she may design a supposedly public building that excludes or limits some members of the public from accessing the building. Whether the occupants of the building *intended* to include persons with physical handicaps is, in some sense, irrelevant if the material design of the building thwarts access to persons with physical handicaps. In similar fashion, a physician’s *intent* to promote a patient’s well-being may be challenged or undermined if the design of best practice advisories prioritizes other interests apart from those of patients, and subsequently constrains or steers physicians’ actions in ways that may be suboptimal for their patients.

To address these challenges, increased transparency in the design process, not treating best practice advisories as the sole source of decision-making information, and communicating the limitations of underlying data and models are good and workable solutions.^9^ But, they are not sufficient. Such approaches are necessary and should be included in any best practice advisory design, revision, or use processes. However, we also argue that deliberate examination of the root causes of potential biases as well as explicit consideration of biases during advisory design, monitoring, and review processes are also necessary.

## ETHICALLY ADDRESSING BIAS IN BEST PRACTICE ADVISORIES

Problematic stakeholder bias occurs when best practice advisory design and modification decisions prioritize other interests over patients’ well-being and therefore are insufficiently oriented to the well-being of patients. In our view, the central, orienting ethical aim of clinical medicine is to serve the patient and prioritize the well-being of patients. If we agree, as so many hospital mission, vision, and value statements attest, that *patients come first*, then as a kind of litmus test, each major best practice advisory design decision should be accompanied by the question: Does this design choice benefit patients *at least as much* or more than other stakeholders?

We propose that explicit ethics-based examination of potential biases occur in best practice advisory design and review processes. In order to sufficiently examine the potential for bias, the first step is to understand the source of such biases. We contend that the primary sources of such biases are the stakeholders themselves. As described in Table 1, each stakeholder is likely to have a vested interest in the outcome of a clinical process, whether that outcome be financial or professional or clinical, and such interests can at times conflict with patient interests. While we acknowledge that not all biases can be removed, and that in some cases bias is perfectly acceptable (eg, bias toward evidence-based practices rather than preference-based practices), bias that results in outcomes that do not benefit patients as much as other stakeholders should be explicitly reviewed, acknowledged, and mitigated where possible.

**Table 1.** Potential stakeholder biases and conflicts of interest

Stakeholder	Potential biases and manifestations of such biases
Hospitals	The needs of the hospital, such as remaining financially viable, are more important than the needs of individual patients. This can manifest via prioritization of meeting quality benchmarks (eg, reduction in readmissions or reduced length of stay) over specific patient health needs, such as by indirectly suggesting that a different diagnosis be used for a readmission
Pharmaceutical, Medical Device, or Diagnostic Companies	Selling products is paramount, as in the Practice Fusion, Inc. case,^2^ and advisories are created that may result in over prescription or overuse of medical services or devices
Physicians	Physicians’ power and authority must be preserved, which may manifest by advising that a certain type of specialist must been seen (or be recommended) or services be provided only by an M.D. (eg, not an advance practice provider) for the benefit of physicians as a whole or a particular specialty rather than for the patient
Algorithms	Algorithms may be biased toward optimization of “rewards” (eg, prevention of high volumes of care), rather than optimization of patient health outcomes or experiences^5^

Mindful of these issues, we argue for inclusion of ethics as one of the core design and evaluation criteria for best practice advisories.^9^ More specifically, we propose that the commitment to the ethical norms of health care should be an explicit consideration in the design, review, and revision process. For example, in our view, explicit consideration of whether or not a best practice advisory design or revision choice benefits individual patients *at least as much* or more than other stakeholders can significantly help to increase recognition of the values being baked into the technology.

Our first recommendation is to explicitly consider potential forms of bias in best practice advisory and review processes. Just as a persuasive justification is often needed in order to gain approval to initially construct a best practice advisory, clear and convincing discussion and documentation of whose interests are served by the design of the best practice advisory, as informed by a stakeholder bias analysis, should be required for implementation. Specifically, each stakeholder in the process should be evaluated for bias by another stakeholder. Then, the final advisory should be evaluated from a patient perspective, even via inclusion of a patient representative where possible, with patient benefits and outcomes taking priority over other stakeholder needs or wants.

Second, we recommend that recipients of best practice advisories take the time, when possible and appropriate, to understand how and why certain actions are being requested or required. Health care providers should be able to identify the benefit to patients in best practice advisories, and if they cannot, then that is a red flag for a potentially deficient or problematic best practice advisory alert. If such critical thinking does not occur initially and as trust in best practice advisories increases, over-reliance may result. Such reliance may allow potential conflicts to persist if relevant questions are not raised at appropriate times. Thus, recipients of such advisories, including physicians and other care providers, are equally responsible for raising concerns when stakeholder biases are potentially present and for raising ethical questions (eg, how does this benefit patients?) in design, review, and revision processes.

Third, we recommend that analytics be applied to best practice advisory use (or non-use) to understand how often such advisories appear and to whom, what types of patients are the most likely recipients, and evaluation of whether or not the advisory was followed as well as the outcome (where possible). In other words, the expected or even ideal outcomes may differ from the actual outcomes and such differences are vital to consider.^10^ For instance, such analysis might reveal that certain physicians are more or less likely to follow the advisory and, of those that do not follow the advisory, perhaps they have reservations about patient benefits (or biases against applying the advice) that should be addressed. It is also possible that the outcomes from the application of the advisory are contrary to or somewhat different than what was expected when the advisory was designed or implemented (eg, volume of prescriptions of a particular drug is higher than expected after a particular advisory appears). Post hoc analysis in this case, even if just simply by reviewing which best practice advisories are currently active in the system, is vitally important. Furthermore, as algorithms play a more dominant role in medicine, it is also possible that such algorithms create or review best practice advisories autonomously, which can result in inadvertent effects if not closely monitored.

Finally, we recommend that a specified individual be charged with ensuring consideration of the “ethics” portion of evaluating a best practice alert. It is our conjecture that IT teams should seek out the opinion of and include an ethicist or those who specialize in ethical analysis on the team. The value of adding an ethics specialist to the team allows for a specific individual to implement a systematic process to evaluate the design of and implementation of the best practice alert. Ethicists are trained to use such processes for other clinical, research, and organizational ethics questions, and inclusion on the IT team will allow best practice advisory (BPA) alerts to get similar benefits. See Table 2 for an example of applying a systematic ethical analysis process to an opioid-based BPA based upon a decision-making process proposed by Nelson.^11^

**Table 2.** Application of an ethical decision-making process^11^

Ethics decision-making steps	Examples
1. Identify the context of an ethical decision	Dependencies on opioids and the potential for over-prescribing has created a need for a BPA
2. Formulate a specific ethical question	Will this opioid-focused BPA be designed to provide *at least as many benefits for patients* as for other stakeholders?
3. Identify stakeholders	Providers Patient Pharmaceutical companies Community
4. Generate options (value promoting and burdens endured)	Implement the BPA for all opioid prescriptions Implement the BPA only when prescribing opioids outside of specified classes Implement the BPA only on opioid prescriptions that exceed a threshold (eg, 10 pills)
5. Select (and recommend) one option	Designing the BPA to trigger only when prescribing specified classes of opioids is ethically justified because it is the only one that benefits patients *at least as much* or more than other stakeholders
6. Discuss how to prevent or address future ethical conflicts	Future classification of opioids may change, creating the potential for new or different ethical conflicts. If such reclassification occurs, ethical considerations for this BPA should be reviewed

Abbreviation: BPA: best practice advisory.

## CONCLUSION

We conclude by restating that best practice advisories have significant potential to increase the efficiency and effectiveness of medicine,^1^ but that problematic stakeholder bias (even if accidental), wherein patient’s interests are not prioritized, must be explicitly considered. Rather than wait for such ethical issues to arise and only reactively address the consequences, we advocate for a proactive identification and mitigation of stakeholder biases in best practice advisories, as well as documentation of whose interests were prioritized and why, in best practice advisory design, review, and revision processes.

## AUTHOR CONTRIBUTIONS

All three authors made substantial contributions to the design, writing, and revising of this work. All three authors approve the final version and are accountable for the content of this article.

## CONFLICT OF INTEREST STATEMENT

None declared.
